# Accelerated Stability Testing of a Clobetasol Propionate-Loaded Nanoemulsion as per ICH Guidelines

**DOI:** 10.3797/scipharm.1210-02

**Published:** 2013-04-07

**Authors:** Mohammad Sajid Ali, Mohammad Sarfaraz Alam, Nawazish Alam, Tarique Anwer, Mohammed Mohsen A. Safhi

**Affiliations:** 1 College of Pharmacy, Jazan University, Jazan, KSA.; 2 Faculty of Pharmacy, Jamia Hamdard, New Delhi-62, India.

**Keywords:** Nanoemulsion, Clobetasol propionate (CP), Shelf life, Stability, ICH

## Abstract

The physical and chemical degradation of drugs may result in altered therapeutic efficacy and even toxic effects. Therefore, the objective of this work was to study the stability of clobetasol propionate (CP) in a nanoemulsion. The nanoemulsion formulation containing CP was prepared by the spontaneous emulsification method. For the formulation of the nanoemulsion, Safsol, Tween 20, ethanol, and distilled water were used. The drug was incorporated into an oil phase in 0.05% w/v. The lipophilic nature of the drug led to the O/W nanoemulsion formulation. This was characterized by droplet size, pH, viscosity, conductivity, and refractive index. Stability studies were performed as per ICH guidelines for a period of three months. The shelf life of the nanoemulsion formulation was also determined after performing accelerated stability testing (40°C ± 2°C and 75% ± 5% RH). We also performed an intermediate stability study (30°C ± 2°C/65% RH ± 5% RH). It was found that the droplet size, conductivity, and refractive index were slightly increased, while the viscosity and pH slightly decreased at all storage conditions during the 3-month period. However, the changes in these parameters were not statistically significant (p≥0.05). The degradation (%) of the optimized nanoemulsion of CP was determined and the shelf life was found to be 2.18 years at room temperature. These studies confirmed that the physical and chemical stability of CP were enhanced in the nanoemulsion formulation.

## Introduction

The aim of the present study was to prove how the quality of the drug product changes with time under the influence of some environmental factors like temperature, humidity, and light that leads to a change in the shelf life of the pharmaceutical product. Nanoemulsions are isotropic, thermodynamically stable transparent (or translucent) systems of oil, water, and surfactants with a droplet size usually in the range of 10–100 nm [1, 2]. They may become unstable due to environmental factors and long-term storage [3–6]. The degree and speed of destabilization vary from system to system. Therefore, it is important for a formulator to carry out studies on the stability of the system. Nanoemulsions in general can be destabilized by the following mechanisms: creaming, flocculation [7], coalescence [8], and Ostwald ripening [9].

Creaming is the separation of emulsion phases based on their marked density difference between the dispersed phase and the dispersion medium. Creaming is an undesirable process; however, it is a reversible process when it is shaken properly. To prevent creaming, the density difference of the dispersed phase and the dispersion medium should not be too high. Other factors which govern creaming according to Stoke’s law are the dispersed phase globule size and the viscosity of the external phase. Flocculation is the reversible association of the dispersed droplets by certain weak bonds that can be easily redispersed through vigorous shaking. Flocculated droplets are characterized by their ability to maintain their shapes and sizes. Flocculation may lead to irreversible coalescence. Systems are stabilized by non-ionic surfactants, so the dispersed phase droplets are attracted to each other by weak van der Waals forces of attraction, but prevented from coalescence by repulsion due to steric hindrance. The van der Waals attractive force between the droplets depends on the radii of the dispersed phase droplets. As the radius of the droplet decreases, the potential attraction also decreases [10]. As the concentration of surfactant increases in the emulsion, there will also be an increase in the thickness of the film formed around the droplets by the surfactant molecules which leads to an increase in steric repulsion, thereby rendering the emulsion to be more stable. As we know, nanoemulsions have a high amount of surfactant and the particle size is very small. So, flocculation may not be an important parameter to be studied in the case of micro- and nanoemulsions. On the other hand, coalescence is the irreversible combination of two droplets to form a larger droplet. Similarly, Ostwald ripening is the irreversible transfer of small droplets into larger ones so as to form new droplets. The Ostwald ripening phenomenon is concerned with systems in which high variation in the droplet sizes is found. Ostwald ripening is the major concern of the stability issue to be studied by formulators for a successful micro- or nanoemulsion system. Coalescence and/or Ostwald ripening phenomena lead finally to the separation of these systems into three phases – oil, water, and the S_mix_ (surfactant: cosurfactant weight ratios). There is another type of emulsion instability which is the phase inversion of the dispersed system. This is characterized by the transformation of the internal phase into the external phase and vice versa.

Stability studies under normal storage conditions can be efficient to find out the stability of the system, however, the time period is a major issue. To generate stability data that are fast and reliable, accelerated stability studies are carried out. Measuring the physicochemical properties of the product under accelerated conditions can reflect the performance of the product over long-term storage. Chemically, a change in pH of the formulation can indicate the degradation or ionization of one or more of the ingredients in the formula. Furthermore, the chemical transformation of the ingredients reflects their incompatibility or degradation which in turn can produce chemically toxic effects in consumers. Besides, conductivity is one of the techniques used to identify the type of emulsion whether it is W/O or O/W. The presence of water in the external phase results in a conductive system. Any inversion in the system can be identified by the measurement of conductivity. Droplet size measurement by transmission electron microscopy (TEM) analysis is a very important factor in assessing the stability of the emulsion system. A change in the droplet size over time is a result of the aggregation and combination of the internal phase droplets to form larger droplets [11]. The viscosity of the system is of high importance in the formation and continuation of the emulsion. A reduction in viscosity over time indicates a kinetically unstable emulsion where the probability is high that freely moving droplets collide with each other and tend to coalesce. Hence, the detection of viscosity changes over time can provide data about system stability [12].

The stability of the emulsion is concerned with the maintenance of the internal phase dispersion in the external phase without having effective changes in both of them. In other words, the system should maintain the same number and sizes of the dispersed droplets in the dispersion media. Clobetasol propionate (CP) is a super high potency dihalogenated corticosteroid used for the treatment of skin disorders such as atopic dermatitis and psoriasis [13, 14].

CP is practically insoluble in water. Hence, the CP nanoemulsion is the O/W type. Therefore, the stability of this system is a critical factor needing to be assessed. Topical corticosteroids have been widely used to treat skin diseases. Their clinical effectiveness in the treatment of psoriasis and atopic dermatitis is related to their vasoconstrictive, anti-inflammatory, immunosuppressive, and antiproliferative effects [15].

CP is used topically as well as systemically in the case of different types of psoriasis. It exerts its action by the inhibition of phospholipase A_2_ which leads to the inhibition of the synthesis of arachidonic acid and controls the biosynthesis of prostaglandins and leukotrienes [16]. However, the clinical limitation of CP is its poor permeability through the skin which reduces its therapeutic effectiveness at the target site. The main limitation lies in the barrier function of the skin. Therefore, the major challenge facing a topical formulation is to provide a sufficient increase in drug penetration into the skin, without any significant functional and histological change in the skin [17].

In recent years, much attention has been focused on lipid-based formulations to improve the permeability and bioavailability of poorly water soluble drug compounds. Many of the dermal vehicles contain chemical enhancers and solvents to achieve these goals. But the use of chemical enhancers may be harmful, especially in chronic application, as many of them are irritants. Therefore, natural oils such as eucalyptus oil are selected, which act as a penetration enhancer as well as a vehicle for the nanoemulsion. Nanoemulsions have been known to increase the therapeutic efficacy of many drugs and also enhance the physical as well as chemical stability of many drugs. Therefore, attempts were made in the present study to enhance the physical as well as chemical stability of CP using a nanoemulsion formulation.

## Materials and Methods

### Materials

Clobetasol propionate was purchased from Mahima Lifesciences Pvt. Ltd. (New Delhi, India). PEG 200, Tween 80, Tween 20, pleurol oleic, glycol, brij35, propanol, isopropyl alcohol, and ethanol were purchased from Merck (Merck, India). Labrasol, safsol, and capryol were obtained as kind gift samples from Gattefosse (Mumbai, India). All other chemicals used were of analytical grade.

### Preparation of Clobetasolpropionate Nanoemulsion

Nanoemulsions of CP were prepared by the spontaneous emulsification method. On the basis of solubility studies, eucalyptus oil, Tween 20, and ethanol were selected as the oil, surfactant, and co-surfactant, respectively. Different combinations of surfactant and co-surfactant were prepared, commonly known as S_mix_. To find out the nanoemulsion region, pseudo-ternary phase diagrams were constructed for each S_mix_ ratio. Finally S_mix_ (1:2) was selected for the preparation of the nanoemulsion. A number of nanoemulsion formulations were prepared based on the different ratio of oil: S_mix_(1:2): water and evaluated for their physical stability studies. Considering the irritation potential of surfactants, the S_mix_ ratio containing a minimum percentage of surfactant was selected for the preparation of the nanoemulsion. The optimized nanoemulsion was prepared by dissolving 0.05% (w/v) of CP in 15% (v/v) eucalyptus oil, then a 35% (v/v) mixture of Tween 20 and ethyl alcohol (1:1 v/v) were added slowly to the oil phase. Then the remaining amount of distilled water was added slowly to get the final preparation of 100% (v/v). A vortex mixer (S0200-21, Labnet International, Inc.) was used for the vigorous shaking of the mixture (oil,S_mix_, and water).

### Characterization of the Nanoemulsion

The morphology and structure of the nanoemulsions were studied using TEM (TOPCON 002B operating at 200 KV and at0.18 nm capable point-to-point resolution). A combination of bright field (BF) imaging at increasing magnification and diffraction modes was used to reveal the form and size of the nanoemulsions and to determine the amorphous or crystalline character of their components.

In order to perform the TEM observations, the concentrated nanoemulsion was first diluted in water (1/10); a drop of the diluted nanoemulsion was then directly deposited on the holey film grid and observed after drying. The emulsion appeared dark and the surroundings bright, and a “positive” image was seen. The direct observation also enabled us to perform selected area electron diffraction (SAED) to check the crystallinity of the nanoemulsion core components [18].

The viscosity of the nanoemulsion was measured using the small sample adapter of a Brookfield rheometer (Model DV-III, Brookfield Engineering Labs., Inc., Stoughton, MA, USA) at 25°C. An average of three data points was obtained to determine the viscosity at a shear rate of 7.34 s^−1^[19].

The refractive index (RI) of the nanoemulsion formulation was determined using an Abbes type refractrometer (precision standard testing equipment corporation, India) [20].

Measurement of the pH of the samples was done by using the pH meter (Cyberscan, Eutech Instruments). Five ml of the sample was transferred into a beaker and the pH meter probe was immersed into the container. Then the pH reading was recorded. The pH meter was calibrated before using it to measure the pH of the nanoemulsion. The pH of the freshly prepared formulation was measured and was used to compare the change in pH of the formulation after specified time intervals at the different temperatures studied.

The conductivity was measured by using the conductometer (Cyberscan, Singapore, Eutech Instruments). An amount of 2 ml of the sample was transferred into a beaker and the conductometer probe was immersed into the bottom of the container. Then the conductivity reading in μs (microsecond) was recorded. The conductivity of the freshly prepared formulations was measured and was used to compare the change in conductivity of the formulation after specified time intervals at different temperatures studied [21].

### Statistical Analysis

Statistical analysis was performed by using the one-way ANOVA test to determine the difference of all the parameters studied initially and after 90 days of observation at all storage conditions. Statistically, a significant difference was considered at a p value of less than 0.05.

### Stability Studies as per ICH Guidelines

Accelerated stability studies were carried out on an optimized CP nanoemulsion according to International Conference on Harmonization (ICH) guidelines. Sufficient replicates of the optimized nanoemulsions were kept at 40°C ± 2°C and 75%± 5% RH. These were placed in a humidity chamber at 40°C ± 0.5°C and 75% ± 5% RH. Samples were withdrawn at 0, 30, 60, and 90 days. The samples were evaluated for droplet size, viscosity, pH, conductivity, and refractive index. We also performed long-term stability studies by keeping the samples at 4°C and 25°C ± 2°C/60% RH ± 5% RH.

For the determination of shelf life, three batches of the optimized formulation were taken in glass vials and were kept at accelerated temperatures of 30°C ± 5°C, 40°C ± 5°C, 50°C ± 5°C, and 60°C ± 5°C for 90 days at ambient humidity (65% RH ± 5%). The samples were withdrawn at regular intervals of 0, 1, 2, and 3 months [22–25].

The RP-HPLC method was used for the estimation of CP in the nanoemulsion formulation. They were chromatographed on a reversed-phase C-18 column (25cm × 4.6mm, 5μ) in a mobile phase consisting of methanol and water in the ratio 80:20 v/v. The mobile phase was pumped at a flow rate of 1.0 mL/min with detection at 241 nm. The detector response was linear in the concentration of 5–40 μg/mL for CP. The intra- and interday variation was found to be less than 1.5%. Retention time was found to be 7.0 min. The proposed method was to find out the concentration of CP in the nanoemulsion formulations at different elevated temperatures. In addition, the sample of pure safsol oil, pure surfactant, pure cosurfactant, and S_mix_ were run separately to check the interference of the excipients used in the formulations [26].

The amount of drug decomposed and the amount remaining (undecomposed drug) at each time interval was calculated. The order of degradation was determined by a graphical method. The degradation rate constant (K) was determined at each temperature. An Arrhenius plot was constructed between log K and 1/T to determine the shelf life of the optimized nanoemulsion formulation. The degradation rate constant at 25°C (K_25_) was determined by extrapolating the value at 25°C from the Arrhenius plot. The shelf life (T_0.9_) of the formulation was determined by using the formula:

$$\text{Shelf life}=\frac{0.1052}{\text{K}}$$

Where K is the degradation rate constant.

## Results and Discussion

Stability studies provide evidence on how the quality of a drug varies over time under the influence of a variety of parameters such as temperature, humidity, and light. An ideal drug product must be fully characterized physically, chemically, and microbiologically at the start of a study and throughout the intended shelf life period. Accelerated stability studies for the CP nanoemulsions were performed according to ICH guidelines for a period of three months. Optimized nanoemulsions were subjected to accelerated stability studies at 40°C ± 2°C and 75% ± 5% RH. The formulation was evaluated on the basis of droplet size, viscosity, pH, conductivity, and RI for the period of three months. The study showed that there was no significant change in all the parameters after three months. The droplet size, conductivity, and refractive index were slightly increased while the viscosity and pH slightly decreased (Table 1).

The stability of the CP nanoemulsions was also checked at refrigerator (4°C) and room temperature (25°C). The study showed that there was no significant change in all the parameters during the 3-month of storage period (Table 1).

These parameters were compared for statistical significance by one-way analysis of variance (ANOVA) followed by the Tukey-Kramer multiple comparisons test using GraphPad InStat software (GraphPad Software Inc., CA, USA). The changes in these parameters were not statistically significant (P ≥0.05). These results indicated that the optimized formulation was stable as there were no significant changes in the physical parameters (droplet size, viscosity, pH, conductivity, and RI). The degradation of CP was very slow at each temperature which indicated the chemical stability of CP in the nanoemulsion formulation. The optimized nanoemulsion was found to be stable chemically as well as physically.

**Fig. 1:**
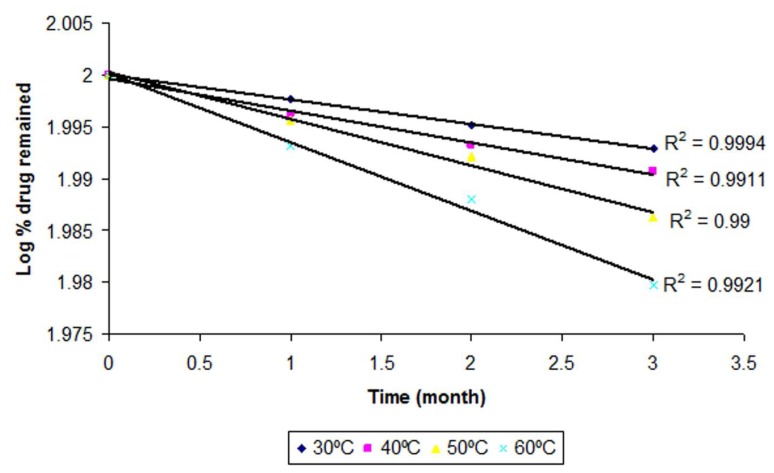
First-order degradation kinetics of CP from the optimized nanoemulsion at different temperatures.

The order of degradation was determined by a graphical method at each temperature. The degraded and remaining concentrations of CP at different temperatures are shown in Table 2. The order of degradation was found to be first-order (Figure 1). The rate of degradation is directly proportional to the first power of the concentration of a single reactant in first-order degradation.

**Fig. 2:**
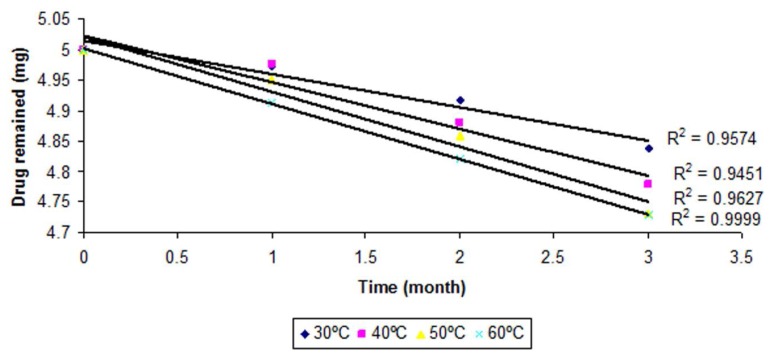
Zero-order degradation kinetics of CP from the optimized nanoemulsion at different temperatures.

From Figure 1 and Figure 2 it was found that the correlation coefficients for the first-order reaction is more reliable as compared to the zero-order, hence the degradation of CP follows a first-order reaction. Therefore, for first-order degradation, the log % of drug remaining was plotted against time (Figure 1) and K was calculated from the slope of the curve at each temperature by the following formula:

$$\text{Slope}=\frac{-\text{K}}{2.303}$$

Where K is the degradation rate constant

**Fig. 3:**
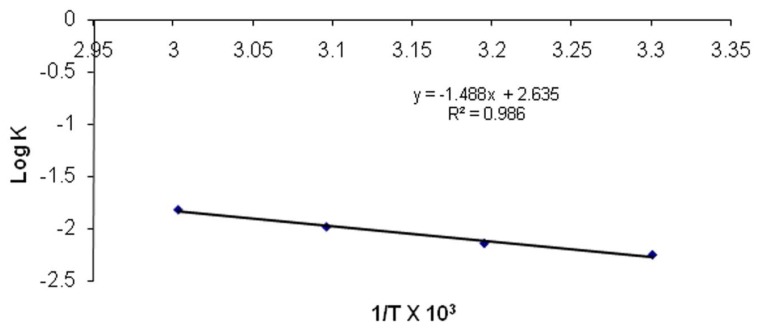
Arrhenius plot between Log K and 1/T for optimized nanoemulsion.

The values of K at each temperature are given in Table 3. The log of drug remaining was plotted against time (months). The slope of each line was obtained and K was calculated by the formula. The effect of temperature on degradation was studied by plotting log K v/s 1/T (Figure 3). The value of K at 25°C (K_25_) was obtained by extrapolation of the plot and shelf life was then calculated. The shelf life of the optimized CP nanoemulsion formulation was found to be 2.18 years.

## Conclusion

The prepared nanoemulsion was stable against creaming, coalescence, phase separation, and Ostwald ripening. It can be concluded that CP, with a very low solubility in the aqueous phase, forms a stable O/W nanoemulsion. Furthermore, the use of a non-ionic surfactant combination can produce a highly stable nanoemulsion preparation. The droplet size, viscosity, pH, conductivity, and RI of the optimized nanoemulsion formulation did not significantly change during the 3-month storage period at different temperatures, suggesting the physical stability of the prepared nanoemulsion. The degradation of CP after three months of storage was found to be lowest in the formulation. The slower degradation of CP indicated the chemical stability of CP in the nanoemulsion. The shelf life of the nanoemulsion formulation was found to be 2.18 years at room temperature (25°C). From this study it can be concluded that the stability (physical and chemical) of CP can be enhanced by incorporating it into a nanoemulsion.

## Figures and Tables

**Tab. 1:** Droplet size, Viscosity, RI, and Conductivity of the optimized nanoemulsion during storage

Time (months)	Temp. (°C)	Mean droplet size (nm) ± SD (n=3)	Mean Viscosity (mP) ± SD (n=3)	RI ± SD (n=3)	pH ± SD (n=3)	Conductivity (μs) ± SD (n=3)
0	4.0 ± 0.5	85.31 ± 0.73	29.41 ± 1.01	1.410 ± 0.024	5.81 ± 0.013	346 ± 2.01
1	4.0 ± 0.5	85.33 ± 0.42	29.38 ± 1.05	1.412 ± 0.028	5.75 ± 0.026	342 ± 2.11
2	4.0 ± 0.5	85.83 ± 0.83	29.34 ± 1.03	1.414 ± 0.025	5.71 ± 0.025	350 ± 2.09
3	4.0 ± 0.5	85.99 ±1.09	29.09 ± 1.07	1.416 ± 0.021	5.69 ± 0.031	351 ± 2.07
0	25 ± 0.5	85.31 ± 0.73	29.41 ± 1.01	1.410 ± 0.024	5.81 ± 0.013	346 ± 2.01
1	25 ± 0.5	85.49 ± 0.93	29.41 ± 1.13	1.412 ± 0.022	5.68 ± 0.021	345 ± 2.27
2	25 ± 0.5	85.28 ± 0.31	29.27 ± 1.35	1.417 ± 0.024	5.61 ± 0.029	350 ± 2.19
3	25 ± 0.5	85.97 ± 0.47	29.03 ± 1.02	1.419 ± 0.029	5.53 ± 0.027	353 ± 2.32
0	40 ± 2	85.31 ± 0.73	29.41 ± 1.01	1.410 ± 0.024	5.81 ± 0.013	346 ± 2.01
1	40 ± 2	85.42 ± 0.63	29.23 ± 1.19	1.414 ± 0.029	5.68 ± 0.015	350 ± 2.47
2	40 ± 2	85.31 ± 0.17	29.17 ± 1.08	1.419 ± 0.041	5.51 ± 0.015	350 ± 1.10
3	40 ± 0.5	86.01 ± 0.69	28.89 ± 1.10	1.424 ± 0.097	5.49 ± 0.015	355 ± 1.52

**Tab. 2:** Degradation of the optimized nanoemulsion

Time (Days)	Temp (°C)	Drug content (mg)	Drug concentration degraded (mg)	%drug remaining	Log % drug remaining
0	30 ± 0.5	5	0	100	2
30	30 ± 0.5	4.972	0.028	99.448	1.9976
60	30 ± 0.5	4.917	0.083	98.87	1.9951
90	30 ± 0.5	4.838	0.162	98.378	1.9929
0	40 ± 0.5	5	0	100	2
30	40 ± 0.5	4.975	0.025	99.128	1.9962
60	40 ± 0.5	4.880	0.12	98.446	1.9932
90	40 ± 0.5	4.778	0.222	97.881	1.9907
0	50 ± 0.5	5	0	100	2
30	50 ± 0.5	4.95	0.05	99.991	1.9956
60	50 ± 0.5	4.86	0.14	98.197	1.9921
90	50 ± 0.5	4.731	0.269	96.894	1.9863
0	60 ± 0.5	5	0	100	2
30	60 ± 0.5	4.912	0.088	98.423	1.9931
60	60 ± 0.5	4.821	0.179	97.274	1.988
90	60 ± 0.5	4.729	0.271	95.433	1.9797

**Tab. 3:** Observation table for the calculation of shelf life for the optimized nanoemulsion

Temp. (°C)	Slope	K×10^−3^ (month^−1^)	Log K	Absolute Temp. (K)	1/T × 10^3^
30	0.0024	5.527	2.25749	303	3.30033
40	0.0031	7.139	2.14634	313	3.19488
50	0.0045	10.364	1.98449	323	3.09597
60	0.0066	15.201	1.81816	333	3.00300
25		4.017	2.39579	298	3.35570
